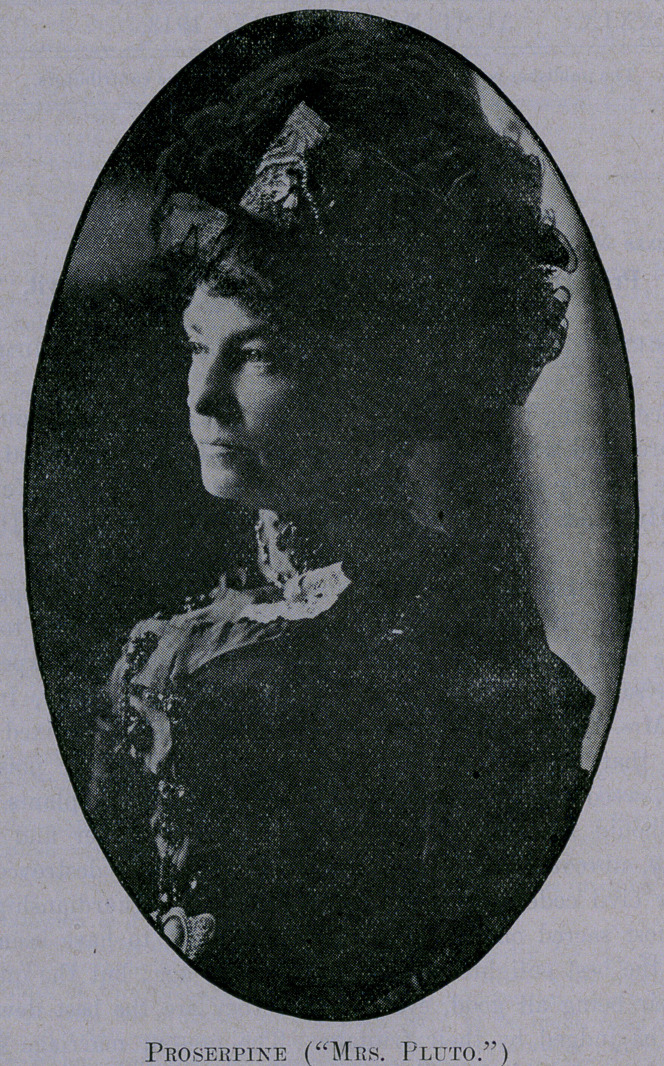# Proserpine (“Mrs. Pluto”) on the Divorce Evil

**Published:** 1913-01

**Authors:** Eloise Shepherd

**Affiliations:** 1902 Hardy street, Houston, Texas


### TEXAS MEDICAL JOURNAL.

Established July, 1885

F. E. DANIEL, M. D., - - - - Editor, Publisher and Proprietor

Published Monthly.—Subscription, $1.00 a Year.

VOL. XXIX. AUSTIN, JANUARY, 1913. No. 7.

The publisher is not responsible for the views of the contributors.

### Original Articles.

For Texas Medical Journal.

### Proserpine ("Mrs. Pluto") on The Divorce Evil.

(COMMENTS ON ARTICLE BY WIFE AND MOTHER IN JUNE NUMBER.)

Since reading the June number of the TEXAS MEDICAL JOURNAL, I've proposed writing what I conceive will be a sidelight, at least, on the leading article for that issue, entitled, "How Can We Hold Our Husbands? or Suggestions for the Correction of the Divorce Evil."

I beg, at the first, that none will understand me as speaking in an unkindly spirit, or in a cruel or critical sense. I, too, am a wife and a mother, qualified thereby to speak from experience for myself if not with authority for others. Personally, I agree with Mrs. Zeralda Wallace, that "divorce is not an unmixed evil." Trees that bear fruit unfit for food, vines that bear poisonous leaves, weeds that choke out the growth of blooming plants are—and should be—selected out from those of a higher and better quality, uprooted, and cast out where they will be destroyed.

Our civil codes—those aiming at established guardianship over the most sacred offices of the race—I concede to have been born from the best enlightenment of the present age; but the question of their being all good, even whether they are the best devisable, must be judged by their fruitage. The present marriage system must be judged before the same tribunal; and unless its fruits rank with the grade of what would constitute the best, then the system should be subject to discussion, at least, and to revision, if need be; such revision as enlightened judgment would offer relative to codes governing in any other department of civilization.

That the Garden of Wedlock is not now a-bloom with life's fairest flowers surely must be plain to every thinker. What, then? Why, revise the statutes, both moral and civil, in so far as practicable, to be sure, and not strive to fit a code to an outgrown, a "tested and tried and found wanting" condition that tells its own story of woe and disaster through the blasted fruits that lie strewn along its wreckage-way.

PROSERPINE ("MRS. PLUTO.")

How? Ah! here we do, indeed, emerge upon grounds whereof we know little as yet. However, no advance can be made unless we pioneer the areas we seek to explore, and earnestly strive to obtain the best guidance for future use.

The writer of the above stated article, who signs herself "A Wife and Mother," I shall designate as "The Writer," in lieu of her name not being given.

She lays down as a major premise, "False Education" as the chief cause of the Divorce Evil; and none can gainsay that proposition. But what constitutes False Education? After all is once said, we students are likely to find ourselves sadly uninformed regarding numerous complex points; but we can only persevere, so let us note this one especially. Wives are admonished to hold their husbands to them "by self-sacrifice, lest they be sacrificed for the 'other woman.'" Will this reasoning bear every test? Let us subject it to a few.

That wives speedily come to loathe the sex-caresses of their husbands is easily understood by wives themselves; and The Writer hits the target straight when she says: "Man has but one nature —he is passionate and aggressive." Well, the same can be stated of the devil, with all righteous respect to him, if devil there be— but is that any argument favoring man's conduct, and seeking to foster his "aggressiveness," when such course proves the wife's utter destruction?

Just here we touch the point where real education is needed to counteract that of a false character. If it be true that "man has but one nature," and that is so carnal it cannot be governed— which present-day civilization would seem to indicate—if his "one nature," I repeat, is so carnal that his most sacred interests— home, wife and children, state, society and himself—must all be sacrificed to it, then it would seem certain he needs some "true education," instead of so much that is false.

In heaven's name! Has man no godly nature at all? Has he none of that soul and spirit character which delights in soulful and spiritual intercourse, but must bend every and all things to the dominion of his corporeal selfhood?

I'm speaking personally, remember, but with all sincerity, when I declare that if I am expected to see any man as a god, or as an Apollo, then he must act that part at least to some degree. On the stage, we determine characters according to the parts they act, and how else, pray, can we see them? How else can we see men? How else can we see human life?

When a man in his home and husbandly estate conducts himself after the manner of a beast, he calls for a tremendous stretch of imagination on the part of his wife when he expects her to see him as a god. I couldn't do it, whether other women could or not. That is for them to determine, fortunately.

A man weds a young girl, as The Writer points out, whose every sex-instinct has been devitalized, crushed, stamped out of existence, so to speak, by false education, truly. And he seems to think that the mere wedding ceremony shall have stricken out all her past life-teachings and transformed her into another creature—one corresponding to himself, dowered by birth with strong tides of sex-desire, and then had that propensity fostered and encouraged by teachers and physicians, and even by society.

True education, as opposed to false, would do more just here toward preventing the divorce evil than all future antidotes, legislative enactments, pulpit, press, school or conventional teachings that could be written or spoken. If he could have been taught to know that with the young wife the sex-nature—weaker in all female animals than in the males at best—must be drawn forth by loving, not by sexual, embraces; that for days, weeks, perhaps, her deeper, her inner, her higher nature must be trained to bloom and fruit toward his desires, the awful tides of crime that now flow through the channel of wedlock would be largely overcome; and then, all through wedded life, true, as opposed to false, education ruling along the same lines, the evil would die out wholly and never flourish again.

A very large majority of wives come swiftly to know that they are to receive none of the refreshing dews of pure love for which their aching, hungry hearts yearn and starve; but that every caress from the husband they love means that same sacrifice to his "one nature." Could he be educated to develop, or to unfold, "another nature," one that would tender to her the heart-food for which she so longs—the caressing, loving, heartfelt affection she so bitterly craves—and tender it without the sex-demands she must teach herself to expect as part of his "one nature," and in receiving, leaves her as starved as before because she realizes she wouldn't have gotten even that semblance of it had she not sacrificed at *his* altar,—could he teach himself this, I repeat, then the "other woman" would forever be his beloved wife, and he would find her so.

It is this unholy sacrifice that extinguishes the Shekinah fires of holy love wherewith God has lighted the halls of woman's heart and endued her with that deathless love which enables her to love on though he sacrifices her, body, soul and spirit, upon the altar of his carnality.

According to my understanding, true sex-education lies somewhat along these lines, and not in seeking to adapt the race to what is proven by "red-light districts" in every city and hamlet on earth to be a destroying evil because of the degrading uses this highest creative function is subject to. I am not aiming to instruct other women in the matter of "holding their husbands." If the husbands make themselves sufficiently "holdable," no such efforts would be conceivable; and that is one of the double rules that apply both ways. When conditions warrant instruction over "how to hold a husband or a wife," then the time has arrived for them to travel in separate paths; for to do otherwise is little else than prostitution in some form or other, however sanctified by marriage or other laws, man-made, as distinguished from God-directed. I would strive to exalt the understanding of man—husbands—in what I conceive to be the "true education" of their own duties in the wedded relation, rather than instruct how to "pull under one yoke" after its galling burden had grown unbearable.

Let me premise: The human trinity has been designated by sacred philosophy as Body, Soul and Spirit. Each entity, or body, we may logically conclude, is endowed with organs—functions, at least—though the organs may not correspond wholly with those we cognize as such in the sense, or corporeal body. The strongest motives, or appetites, the desires, say—or whatever term best suits in naming them as they pertain to the body or corporeal self—are carnal, or what Saint Paul calls "the lusts of the flesh." The passions are of the Soul, wherein we speak of being passionately fond of music, art, or whatever. Love, pure, imperishable, deathless—that principle which lives on and on through endless eternities—is the highest, the innermost, the sacred God-in-us; and who believes it ever was "God-in-us" when it turns to loathing and disgust?

Now, in this matter of True Education: Why cannot man learn what woman already knows, namely, that just according to the inner, the higher, grade of activity in sex-life, just upon that plane where *man* and *woman* in soul and spirit meet, there is to be found and enjoyed the highest, the holiest and most transcendent bliss?

"Oh, you can't teach a man that!" is declared when attempting this teaching. And what can more thoroughly prove his need of this very teaching? Must woman in her divinity be sacrificed because he will not learn higher truths concerning himself and the race? Must the feminine half of creation and his own posterity be doomed to dwell upon a plane of corporeality, when the gates of wisdom swing wide for his entrance into schools where a diviner knowledge, a higher wisdom and more transcendent joys await him? I am among those who believe that unless man, as the exemplar of God in teaching His holy truths, is willing to promote somewhat of his own fleshly conduct to the plane of godliness he teaches to others, then he should not be aided and abetted in fostering a deadly propaganda about his "one nature" and its "aggressiveness."

My nature is "aggressive," too—so aggressive with soul-hunger that all my years have been consumed in the deep heart-cry for loving, sympathetic companionship on the soul and spiritual planes; and, moreover, had I been so enslaved as that I was doomed to remain on the sensual plane of soul-starvation, with no hope of escape or relief, I should have been driven to madness or sought retreat in the grave long since.

Now, when the passions, the soul-desires, of a woman's nature are as "aggressive" as that, who is able to teach me that it is not a husband's duty to meet them with food for their own peculiar needs, as well as to continually demand sacrifice from me—sacrifice from a vessel he has long emptied, and done nothing toward filling afresh?

That a woman comes to "loathe" the advances of her husband cannot be wondered at, when physiological laws alone are invoked. No matter how great "the appetite for some delicious and wholesome food" may be, once that need, that desire, is satisfied, the call no longer exists. But suppose the stomach is then forcibly fed and overfed with the same "sweet morsel"; no opportunity afforded for a return of the appetite is allowed, but constant "feeding" of the same food is thrust upon the eater; and what else than a sickened and nauseated stomach can be expected? So it is with that higher, holier and more complex stomach where the food—the bread of life, namely: pure love—is digested. That the supply is sufficiently abundant for a hundred feeders is no reason why it should all be expended upon one.

Palliating an evil, painting it over with holiness, trying to see it as something godly, for the sake of "holding" the cause, no matter what the condition whence it arose, seems to me what the Savior meant when He cautioned His students against building their houses on the sand; for a sandier, a less stable, foundation than that outlined by The Writer I cannot conceive. I may be in error, but I repeat, this is the viewpoint from which I must conclude a study of years. Insanity, crime, disease, all are increasing alarmingly. The cause lies somewhere; and if the marriage institution as instituted by mankind is right in its every aspect—while out from its abode flows this terrible stream of sin—where shall we seek for the hidden springs of all this woe wherefrom the race is suffering? I am open to conviction. Let some abler student point the truth to me, and I'll swing into the ranks of the better reformers.

Of course, this kind of True Education will not educate the masses—at least, the masses of men. Of that I am well assured; but that does not affect its logic, its truth. Sex-appetite on the part of the wife grows out of expressions of love on the part of the husband, unaccompanied by demand; and whoever among the husband-army doubts this "truly medical, or remedial," suggestion, let him simply try it. *He* doesn't need this fostering of appetite, for it seems to be natively in a "fostered" state; but let him commence offering his wife the small courtesies, the attentions and devoted, loving thoughtfulness he exercised toward her during courtship; and if he continues this course for a reasonable time—time enough to germinate, grow and blossom this inner, this divine nature in woman—he will soon see her meeting him, not with a sham pretense of enjoying him "as a god," when such a thing is impossible in reality; but he will find that up from her heart, from her soul, from the depths of her heavenly being wells a true response to him in the realm of his own better efforts. And, together with that consciousness, will have sprung up a different and better happiness in his own higher nature—a condition that won't need eternal fertilizing to keep active, but a self-renewing estate, founded upon truth instead of error. He will find this latter triumph yielding him a deeper, and holier, and mightier happiness than he had ever before drawn from the well-springs of fleshly being.

I am not in favor of sterilizing the unfit. Unless the tide of sense can be controlled, then full emasculation would be better for the race. Such action, without the accompanying Education I am striving to impress, would only add to the awful tide of abuse that is now driving women to asylums and to their graves. Without being taught the diviner side of matrimonial love, while rendered incompetent to procreate, only a fiercer desire and a still fiercer gratification would result, placing an added burden upon a womanhood already groaning and dying beneath the terrible strain of overindulged sex natures.

A further education along these lines may be suggested from the fact stated by The Writer of man's sex-natures increasing in strength after the period of 40 to 60 years of age, while woman's spiritual and intellectual natures grow stronger during the same period. What, pray, is education, unless it educates? Man could turn his best energies toward higher things after this period equally as well as woman. Arguing otherwise is rating men as possessing the lesser, or at least the weaker, intellect; and we opine that few of them will accept such verdict.

The contribution, "Should Sex Hygiene Be Taught in the Home and School?" by Dr. Frederick Eby of the University of Texas, in the September issue, is most excellent, and to it I would reply: *"Sex, both as to hygiene and philosophy, should be taught wherever there is an ear to hear."* We may not all cognize the fact as I am impelled to state it, but Sex, in its highest and innermost sense, is *the Creator—God.* I speak it reverently. I don't mean the fleshly organs, nor even the sense-functions of sex, but I do mean *Sex,* the creative energy, the incomprehensible, the mighty, the indescribable, the unspeakable, the well-nigh unthinkable *Force* that moves, controls and creates all things. God, as the Creator, when adapting Himself to this objective plane, differentiated Himself into Man and Wo(mb)-man; into the Positive and Negative; into the Bestower and the Receiver. Out from this differentiation—then the differentiation *in righteous union*—He designed that a race should evolve wherein He, the Highest Principle conceivable *to the plane,* would be glorified. But can this design of the Almighty be realized so long as His functions are debased to selfish appetites? I answer, No!—a thousand times No! No! No!

*God is good.* Truly! But because God is good it dare not be conceded that the divine, the godly, the most glorious attribute in humanity may be devoted to the lowermost, the corporeal, the mere fleshly appetites, to the exclusion—not to say destruction—of the highermost, the spiritual, the superhuman. Once teach mankind that complete dominion over carnality precludes the necessity for "sterilizing" and elevates the entire being far above the plane of sensuality into one of pure spirituality, thus placing him upon a height where more transcendent functions operate to a more perfect, a more exalted, enjoyment, expressing the better selfhood through a glorified being here and now, instead of leaving in the wake of carnal fires unchecked, nothing save cinders and blackened death, and surely this Education will endue him with a desire for such wisdom and strength and power as redeems and makes the creature godly, as the Creator is God.

Conservation of Force, or Energy, is regarded a comparatively recent discovery, as we count centuries. But the principle of keeping our possessions if we wish to have them is as old as life expression.

A university is being considered in this country—and it is in Texas, too—in which there is to be established a "chair of eternal life." Is that startling? Life, life essentially, is eternal; and may we not seek eternal proofs or instances of it?

So long, however, as that organized quantity we cognize as one human being fails to conserve his life, but chooses to dissipate it after the impulses of appetite, so long will any demonstration remain undemonstrated.

Invoking some law or introducing some agency whereby "to hold our husbands" would be eminently righteous and holy, were that law or agency drawn from or sought for in realms divine—sought in heavenly and not earthly conditions and actions. Needs the sun exert any kind of "means" whereby to "hold" the earth in her orbit? The absurdity of seeking for such "means" is patent in itself. Attraction is the one, the supreme law; and unless it operates of its own volition, then any attempt at originating superficial efforts must stamp themselves as false; hence failures in performing the very purposes we seek. Man and woman attract each other through love divine, and not through senses human—except as love divine expresses through senses human—and when that is the case, no efforts at "holding" each other are necessary.

Again I ask that no one will feel that I have tried to specially oppose their deductions. Unless I used those employed by former writers in an effort to do just what I am trying to accomplish, namely, to make just a little clearer the way to a higher and better life, I couldn't point the "points" I wished. I ask that The Writer especially entertain no sense that I have tried to upset her theories. I cognize all too gravely the need of "holding" the provider, the father, the husband; but I *do not* believe in sacrificing principle to practice, and—again I speak for myself—I'd prefer going into some one's kitchen and washing dishes till my fingers wore off to the middle joint than permit my own person to be defiled by that which, unless accompanied by purest love-impulses, appears to me no less than prostitution made into a form of respectability through codes accepted by society. Adieu for this time.

ELOISE SHEPHERD,
1902 Hardy street, Houston, Texas.

## Figures and Tables

**Figure f1:**